# Novel Chronic Anaplasmosis in Splenectomized Patient, Amazon Rainforest

**DOI:** 10.3201/eid2808.212425

**Published:** 2022-08

**Authors:** Olivier Duron, Rachid Koual, Lise Musset, Marie Buysse, Yann Lambert, Benoît Jaulhac, Denis Blanchet, Kinan Drak Alsibai, Yassamine Lazrek, Loïc Epelboin, Pierre Deshuillers, Céline Michaud, Maylis Douine

**Affiliations:** Centre National de la Recherche Scientifique, Montpellier, France (O. Duron, R. Koual);; MIVEGEC, Montpellier (O. Duron, R. Koual, M. Buysse);; Institut Pasteur de la Guyane, Cayenne, French Guiana (L. Musset, Y. Lazrek);; University of Montpellier, Montpellier (M. Buysse);; Centre Hospitalier Andree Rosemon, Cayenne (Y. Lambert, L. Epelboin, M. Douine);; University of Strasbourg, ITI InnoVec, and Hôpitaux Universitaires de Strasbourg, Strasbourg, France (B. Jaulhac);; Centre Hospitalier de Cayenne, Cayenne (D. Blanchet, K. Drak Alsibai, L. Epelboin C. Michaud);; Ecole Nationale Vétérinaire d’Alfort, Maisons-Alfort, France (P. Deshuillers)

**Keywords:** anaplasmosis, Amazon rainforest, bacteria, vector-borne infections, zoonoses, French Guiana

## Abstract

We report a case of unusual human anaplasmosis in the Amazon rainforest of French Guiana. Molecular typing demonstrated that the pathogen is a novel *Anaplasma* species, distinct to all known species, and more genetically related to recently described *Anaplasma* spp. causing infections in rainforest wild fauna of Brazil.

Anaplasmoses are emerging tickborne zoonoses caused by intracellular bacteria of the *Anaplasma* genus. In total, 8 *Anaplasma* species and several candidate species have been described, including at least 5 species infecting humans (*1*,*2*). Of particular concern, the agent of human granulocytic anaplasmosis, *A. phagocytophilum*, has a specific tropism to polymorphonuclear neutrophils (*1*,*3*). Another species, provisionally named *A. capra*, recently described from asymptomatic goats, is now recognized as an agent of human intraerythrocytic anaplasmosis in China (*4*). The 3 other species detected in humans are major veterinary agents sporadically identified in few patients worldwide: *A. ovis* and *A. bovis* in erythrocytes and *A. platys* in platelets (*1*,*5*). Human anaplasmosis are consistently associated with persons who live in rural areas in habitats favorable to ticks or who work closely with domestic animals (*1*,*6*). However, recent surveys report the presence of novel *Anaplasma* species of undetermined zoonotic potential in wild fauna (*1*,*2*).

## The Study

We assessed the presence of *Anaplasma* in blood samples of clandestine gold miners working in the Amazon rainforest of French Guiana. This 83,000 km^2^ territory, located between Suriname and Brazil, is one of the regions of highest biodiversity in the world, with >280 species of wild mammals (*7*). The human population of French Guiana (≈284,000 inhabitants) is concentrated principally in a handful of towns spread along the coastline and the 2 main rivers (*8*). The interior is largely uninhabited and covered by dense rainforest, where illegal gold mining camps are located (*9*,*10*).

We examined 363 archived DNA extracts obtained from human blood samples. We primarily collected these samples in 2019 as part of Malakit, a malaria survey in remote mining camps in French Guiana (*11*). To characterize the whole bacterial diversity, we typed DNA blood samples by using a high-throughput bacterial 16S rDNA (*rrs*) sequencing approach (bacterial barcoding) (*12*). Bacteria were characterized as operational taxonomic units (OTUs) and amplicon sequence variants (ASVs) and taxonomically assigned by using the Silva database (https://www.arb-silva.de).

Examination of OTUs and ASVs revealed the presence of *Anaplasma* sequences in 1 DNA sample. No OTU or ASV assigned to the *Anaplasma* genus or to the Anaplasmataceae family was detected in the 362 other samples. We further conducted 2 independent *Anaplasma*-specific PCRs targeting a region of the 16S rDNA gene (544 bp) and the 23S–5S (ITS2) intergenic region (423 bp) using techniques described by Calchi et al. (*13*) and obtained amplicons of correct sizes for the positive sample. The Sanger sequencing of amplicons obtained with each pair of primers confirmed the presence of *Anaplasma*. These sequences have been deposited to GenBank (accession nos. ON513878, ON521229).

We used BLAST (https://www.ncbi.nlm.nih.gov/blast/Blast.cgi) to compare the 16S rDNA and ITS2 nt sequences with *Anaplasma* sequences available in GenBank. None of the nucleotide sequences observed in this study are 100% identical to known *Anaplasma* sequences. The 16S rRNA sequence showed highest identities with *Anaplasma* found in wild fauna of Brazil, including an *Anaplasma* sp. detected in *Amblyomma coelebs* ticks collected on South American coatis, *Nasua nasua* (99.8%; GenBank accession no. MT019560); another *Anaplasma* sp. of black rats, *Rattus rattus* (99.8%; GenBank accession no. KY391803); and *Candidatus* Anaplasma amazonensis (*13*) of brown-throated sloths (*Bradypus variegatus*) and two-toed sloths (*Choloepus didactylus*) (99.1%; GenBank accession no. MT199827). All other *Anaplasma* species showed identities <99%. The ITS2 sequences showed highest nucleotide identity with *Candidatus* A. amazonensis of sloths (96.8%; GenBank accession no. MT267354) and lower identities with other *Anaplasma* species or strains (<92%). On account of these distinct genetic traits, we propose the designation *Candidatus* Anaplasma sparouinense for this novel bacterium. The specific name refers to the Sparouine River, where the infected patient lived.

**Figure 1:**
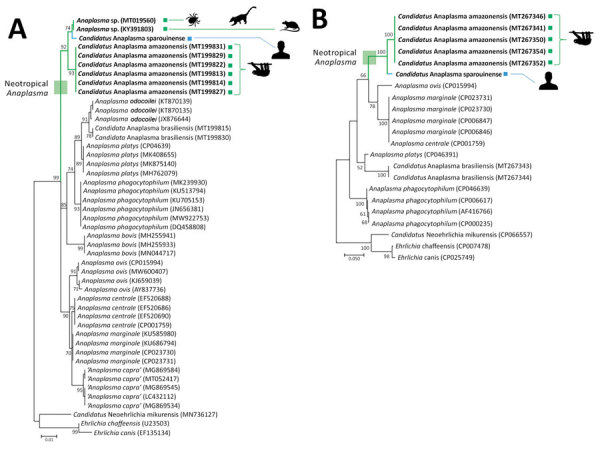
*Anaplasma* phylogenies for the *Candidatus* Anaplasma sparouinense species characterized from French Guiana and reference sequences. Trees were constructed by using maximum-likelihood estimations based on best-fit approximation for the evolutionary model Hasegawa-Kishino-Yano plus invariant sites for 16S rDNA with 485 unambiguously aligned bp (A) and ITS2 sequences with 387 unambiguously aligned bp (B). Bold indicates *Anaplasma* species and strains specific to the Neotropics. GenBank accession numbers of sequences used in analyses are shown on the phylogenetic trees. Numbers at nodes indicate percentage support of 1,000 bootstrap replicates. The scale bar is in units of substitution per site.

We conducted phylogenetic analyses on the basis of these 16S rDNA and ITS2 nucleotide sequences by using the maximum-likelihood method. We obtained trees of similar topologies with a robust clustering of *Candidatus* A. sparouinense with some *Anaplasma* associated with Brazilian wild fauna: *Candidatus* A. sparouinense is phylogenetically related to *Anaplasma* sp. infections detected in ticks of coatis, black rats, and, to a lesser extent, to *Candidatus* A. amazonensis of sloths (Figure 1). Altogether, they delineate a clade of neotropical *Anaplasma* divergent to all other *Anaplasma* species (Figure 1).

The DNA sample positive for *Candidatus* A. sparouinense was from a 58-year-old man who had a history of posttraumatic splenectomy and malaria attacks caused by *Plasmodium vivax*. This patient originated from Maranhão, Brazil, but had been working exclusively in the rainforest of French Guiana for the past 3 years. The Sparouine anaplasmosis was retrospectively diagnosed in September 2021 on the basis of PCR survey of previous blood samples (October 2019 and May 2021) and blood smears (October 2019).

**Figure 2:**
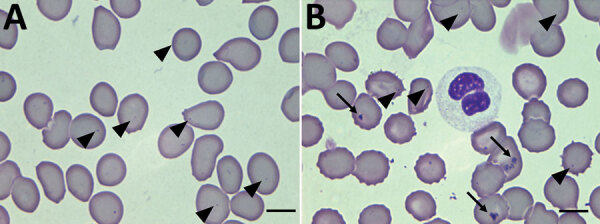
Thin films of a blood sample collected in October 2019 from a patient in French Guiana. Inclusions of *Candidatus* Anaplasma sparouinense are located at the periphery of the red blood cells as small round dots of 0.3–0.4 µm (arrowheads). Other red blood cells contain Howell-Jolly bodies of various shapes and sizes >1 µm (arrows). Some Howell-Jolly bodies are found in the background of the smears. Wright-Giemsa stain; original magnification ×100.

We primarily detected the presence of *Candidatus* A. sparouinense in a blood sample collected in October 2019. At that time, the patient was asymptomatic, including no fever and blood pressure at reference levels; tests were negative for agents of diseases usually tested for in French Guiana (serologic assays for yellow fever, Q fever, hepatitis B and C, HIV, and syphilis and molecular tests for malaria and leptospirosis). He displayed anemia, a hemoglobin level of 10.5 g/dL. The reexamination of Giemsa-stained thin blood film taken for malaria diagnosis at that time revealed the presence of intraerythrocytic bodies, which could be *Candidatus* A. sparouinense. No infection was detected in granulocytes and platelets, but around one third of erythrocytes harbored 1 or 2 small, round, dark purple inclusions located at their periphery, which could be *Anaplasma* (Figure 2). We also detected the presence of Howell–Jolly bodies in erythrocytes (Figure 2, panel B), which could be a consequence of splenectomy.

Eighteen months later (May 2021), the patient was admitted to the Cayenne Hospital Center with fever, myalgia, headache, epistaxis, and severe anemia (hemoglobin 6.6g/dL). A broad microbiologic investigation ruled out COVID-19, dengue, chikungunya virus, Zika virus, influenza, malaria, HIV, hepatitis B and C, and leptospirosis. The only positive test was a subnormal level of *Coxiella burnetii* IgM and IgG (phase II IgG 64, IgM 96; phase I negative), which led to the introduction of antibiotic treatment (doxycycline 100 mg 2×/d for 21 d and ceftriaxone 1 g/d for 5 d). The anemia was considered autoimmune hemolytic because of a positive Coombs test and was thus treated with prednisolone with decreasing doses from 60 mg/day to 10 mg/day for 3 months. The patient recovered within 3 weeks; symptoms resolved, and his hemoglobin level improved to 9.4 g/dL at discharge. Our a posteriori *Anaplasma* PCR survey of May 2021 blood samples (before and at day 7 of antibiotic treatment) again revealed the presence of *Candidatus* A. sparouinense. No further blood sample was preserved; thus, the disappearance of the *Anaplasma* infection at the end of antibiotic treatment could not be confirmed.

## Conclusions

We characterized *Candidatus* A. sparouinense as a novel human intraerythrocytic pathogen. The infection arose over at least 18 months in a patient living in the rainforest of French Guiana who was potentially more susceptible because of a previous splenectomy. The phylogenetic proximity of *Candidatus* A. sparouinense to other *Anaplasma* associated with Amazon ticks and wild mammals highlights that a genetic cluster of *Anaplasma* is circulating in French Guiana and Brazil. These neotropical *Anaplasma* species might represent a source of novel infections to humans. Better documentation of the diversity and transmission cycles of *Anaplasma* in the Amazon rainforest is needed, as recently highlighted for other novel tickborne pathogens described in French Guiana (*14*,*15*).
